# Supplementary material for: AFB1 Triggers Lipid Metabolism Disorders through the PI3K/Akt Pathway and Mediates Apoptosis Leading to Hepatotoxicity
Source: Foods. 2024 Jan 3;13(1):163. doi: 10.3390/foods13010163 (PMC10778638; doi:10.3390/foods13010163)
Supplement: Supplementary file 1 [file foods-13-00163-s001.zip › foods-2768502-supplementary.pdf]

Supplementary Materials

Catalog

**Test S1**	Experimental methods for lipidomics analysis
**Figure S1**	Survival analysis of core genes in the TCGA-LIHC cohort (A) and their expression in GEO datasets (B)
**Figure S2**	The dose–response curves of cell viability caused by AFB1 in HepG2 cells as determined by CCK-8 assays
**Figure S3**	Chromatographic lipid profiles of HepG2 cells in positive and negative ion modes
**Figure S4**	Evaluation of QC samples in positive and negative mode
**Figure S5**	The evaluations of the non-targeted lipidomics OPLS-DA models in the comparison between the control group and AFB1 exposure groups
**Table S1**	Detailed information of differential expression lipids

Test S1. Experimental methods for lipidomics analysis.

Frozen cell samples were thawed on ice and broken by an ultrasonic cell pulverizer. The extraction process of intracellular lipids was performed following the traditional Folch method with minor modifications. Briefly, 300 μL methanol–dichloromethane (2:1, v/v) was added to 100 μL cytosol. The mixture was vortexed for 30 s and centrifugated at 10000 r min −1 for 5 min. Afterward, the supernatant solution was transferred to a glass tube. Subsequently, the same extraction process was repeated to increase the extraction efficiency. The extracts were combined in a glass tube. Next, the extracts were evaporated to dryness under nitrogen and finally resuspended in 0.5 mL methanol for lipid analysis. Equal quantities of analytical samples were blended for the preparation of quality control (QC) samples to evaluate the stability of the instrument and determine data reliability.

Lipidome profiling was carried out using LC-IM-MS instrumentation (ACQUITY UPLC with a SYNAPT G2-XS IM-Quadrupole Time of Flight (QTOF) Mass Spectrometer (Waters Corporation, Milford, MA, USA)). Data were collected by scanning in the resolution mode and MSE mode from 50 to 1200 m/z with a scan duration of 0.8s. Two channels of MS data were recorded in the MSE mode, one with a low collision energy (4 eV) and the other with a collision energy ramp (15–50 eV) to obtain fragmentation data. For accurate mass determination, leucine enkephalin was utilized as the lock mass (reference mass) and the device was calibrated with sodium formate. Ion mobility spectroscopy was carried out using nitrogen drift gas and calibrated with Major Mix infusion. The source parameters were as follows: ion source type, ESI source; scan mode, positive (+)/negative (−) ion mode; capillary voltage, 2.5 kV (+)/2.0 kV (−); source temperature, 100°C; and sampling cone voltage, 40 V. The autosampler tray was set to 8 °C. Lipid extracts (2 μL injection volume) were separated by a CSH C18 column (Waters 2.1 ×150 mm, 1.7 μm), kept at 50 °C. A binary solvent system of mobile phase A (acetonitrile: water (6:4, v/v)) and mobile phase B (isopropanol: acetonitrile (9:1, v/v)) was used, with both phases containing 10 mM ammonium formate and 0.1% formic acid. Separation was carried out with a flow rate of 0.28 mL/min, for a total of 32 min, with the following gradient: 0 min, 60%A; 4min, 55% A; 4.1 min, 50% A; 12 min, 44% A; 12.1 min, 30% A; and 26 min, 1% A; followed by 6 minutes of re-equilibration to 60% A.

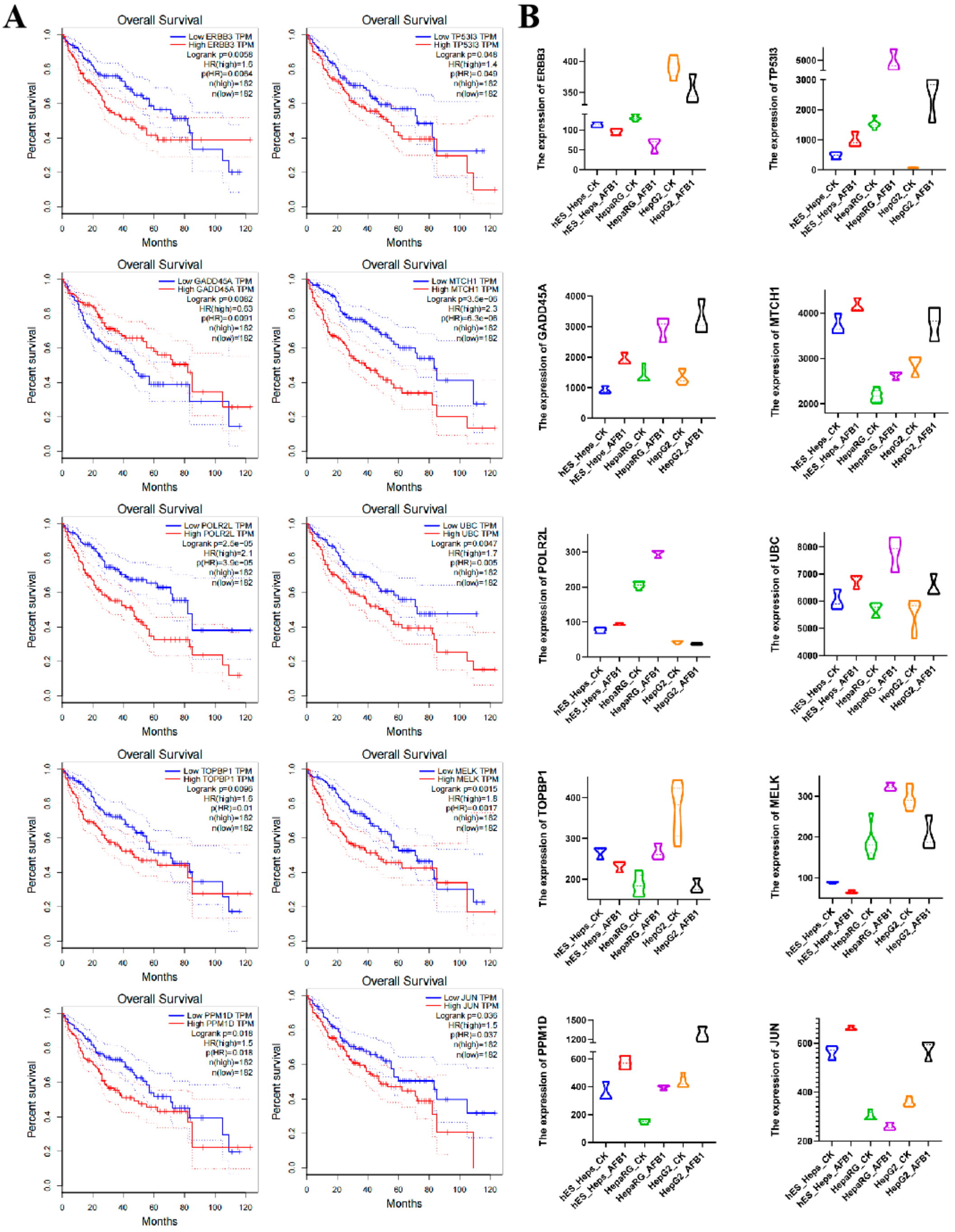

Figure S1. Survival analysis of core genes in the TCGA-LIHC cohort (A) and their expression in GEO datasets (B).

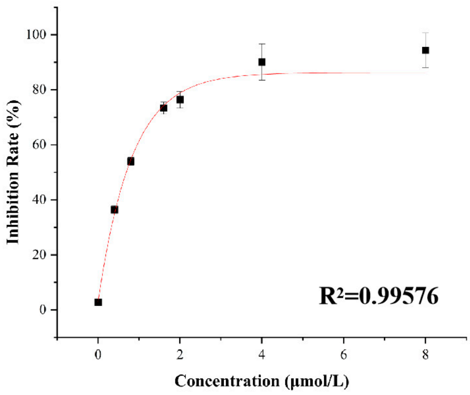

Figure S2. The dose–response curves of cell viability caused by AFB1 in HepG2 cells as determined by CCK-8 assays.

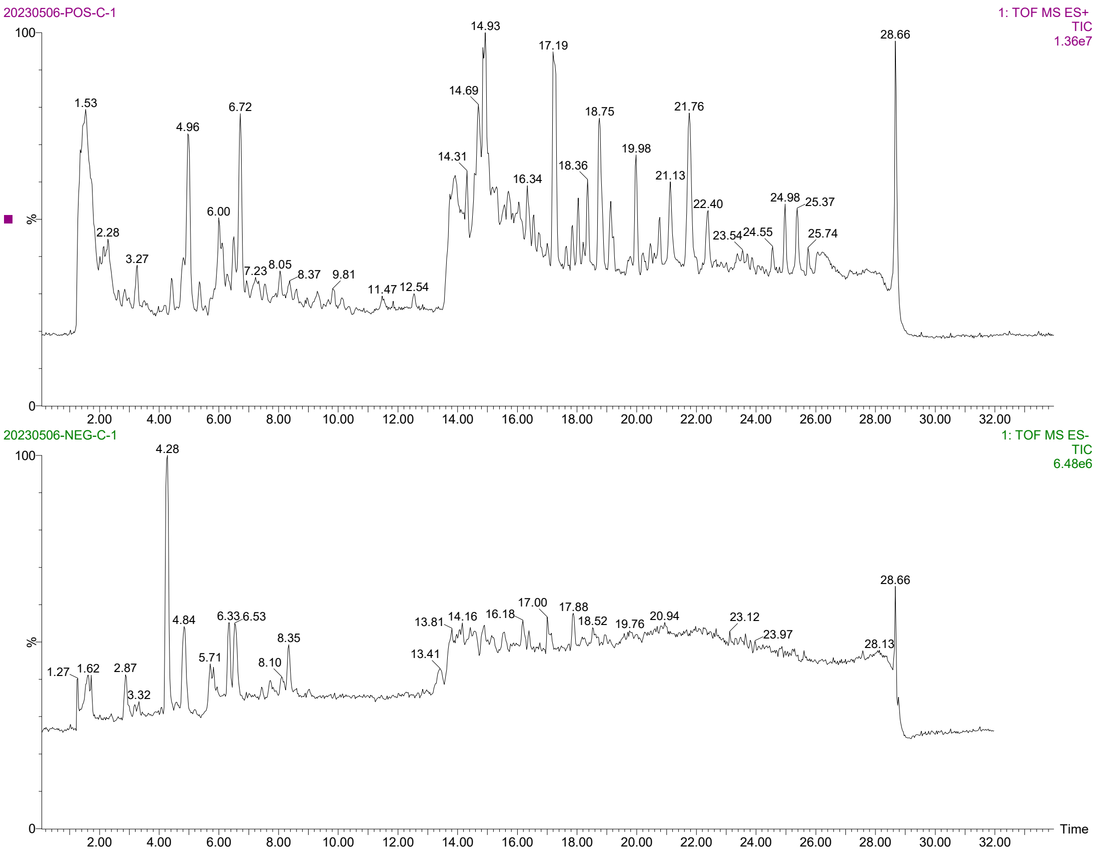

Figure S3. Chromatographic lipid profiles of HepG2 cells in positive and negative ion modes.

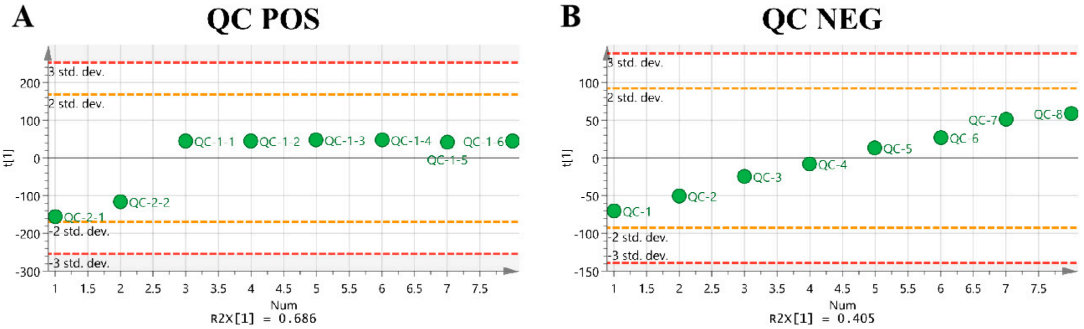

Figure S4. Evaluation of QC samples in the positive and negative mode.

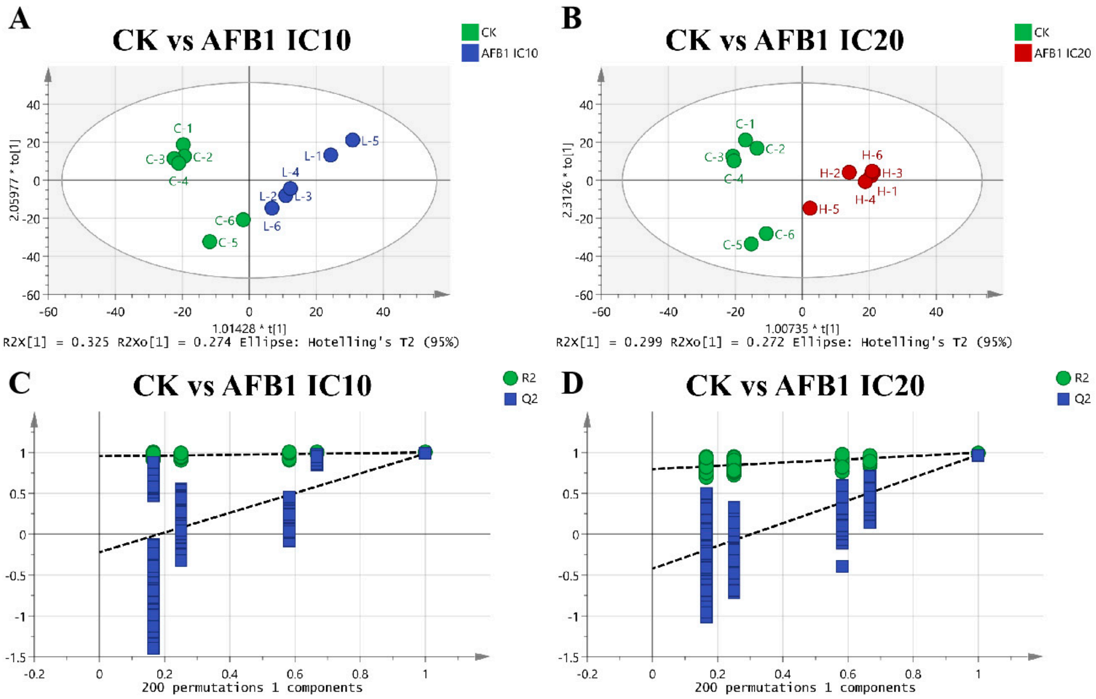

Figure S5. The evaluations of the non-targeted lipidomics OPLS-DA models in the comparison between the control group and AFB1 exposure groups. (**A**) Comparison between the control group and the IC_{10} group: intercept values of R2 and Q2 were 0.955 and -0.22, respectively; (**B**) Comparison between the control group and the IC_{20} group: intercept values of R2 and Q2 were 0.797 and -0.422, respectively.

Table S1. Detailed information on the differential expression lipids.

Metabolites	Compound ID	Class	Sub Class	CK VS AFB1 IC10			CK VS AFB1 IC20		
				P-value	FC	VIP	P-value	FC	VIP
Dihydrocycloartomunin	10072454	PK	PK	0.03	0.36	1.67	0.03	0.34	1.37
Angoluvarin	639275	PK	PK	0.03	0.13	1.54	0.02	0.09	1.45
BMP 16:3_20:5	138217136	GP	BMP	0.03	0.31	1.6	0.01	0.21	1.64
LPG 20:2	138119475	GP	LPG	0.01	0.18	1.82	0.02	0.21	1.61
NA 18:2;O3	LMFA08020334	FA	NA	0.04	0.05	1.43	0.03	0	1.4
FA 34:6;O2	122490529	FA	FA	0.02	0.05	1.59	0.02	0.04	1.49
SL 15:3;O/16:3	164263236	SL	SL	0.04	0.03	1.44	0.04	0.02	1.35
SM 20:1;2O/12:0	138237213	SP	SM	0.01	0.05	1.68	0.03	0.13	1.37
cis:corossolone	11093061	PK	PK	0.02	0.26	1.42	0.04	0.32	1.55
PE:Cer 16:1;2O/26:7	165198142	SP	PE-Cer	0.03	0.12	1.5	0.03	0.07	1.52
BMP 10:0_20:1	138154018	GP	BMP	0.03	0.1	1.52	0.02	0.06	1.5
DLCL 20:0_24:1	165197585	GP	DLCL	0.03	0.13	1.53	0.03	0.13	1.5
Myxol 2':methyl hexoside	162910692	PR	PR	0.03	0.04	1.54	0.03	0.05	1.49
ST 24:1;O4;G/20:4	164497435	ST	ST	0.03	0.5	1.25	0.05	0.44	1.5
Cer 14	164230884	SP	Cer	0.05	0.17	1.4	0.03	0.08	1.51
PA 38:8	52928981	GP	PA	0.02	0.03	1.58	0.02	0.04	1.48
PE 16:0_22:6	6426738	GP	PE	0.02	0.1	1.6	0.01	0.01	1.62
PI 39:1	LMGP06010241	GP	PI	0.03	0.07	1.47	0.02	0.01	1.44
PC O-24:6_11:0	165185238	GP	PC	0.02	0.07	1.6	0.02	0.01	1.58
SM 34:3;2O/3:0	164283258	SP	SM	0.03	0.12	1.59	0.01	0.02	1.67
PG O-26:7_11:0	165241756	GP	PG	0.03	0.13	1.54	0.01	0.01	1.63
Delphinidin 3:(6'':malonylglucoside):5:malonylglucoside	11972331	PK	PK	0.02	0.07	1.64	0.01	0	1.66
SHexCer 17:1;2O/20:2	138185909	SP	SHexCer	0.03	0.14	1.53	0.01	0.01	1.63
DGDG O-11:0_18:5	164308181	GL	DGDG	0.02	0.09	1.62	0.01	0	1.66
Volicitin	10960832	FA	FA	0.05	0.2	1.44	0.01	0.04	1.65
ADGGA 22	138196138	GL	ADGGA	0.04	0.23	1.43	0.03	0.17	1.55
MGDG O-26:2_28:7	164447231	GL	MGDG	0.04	0.07	1.48	0.03	0.02	1.52
PC 11:0_26:6	164242650	GP	PC	0.04	0.16	1.52	0.01	0.02	1.66
LNAPS 17:2/N:24:2	165226409	GP	LNAPS	0.01	0.02	1.74	0.01	0	1.69

PC O-17:2_22:6	165321844	GP	EtherPC	0.02	0.08	1.64	0.01	0.01	1.65
Cer 23:0;2O/13:0	138281970	SP	Cer	0.02	0.03	1.66	0.02	0	1.61
ST 27:2;O6;Hex	101514326	ST	ST	0.04	0.02	1.51	0.04	0	1.43
MGDG O-28:2_7:0	164214536	GL	MGDG	0.02	0.05	1.7	0.01	0.01	1.67
NAGlySer 26:7/19:2	164300449	FA	NAGlySer	0.02	0.1	1.64	0.01	0.03	1.67
PS O-23:0_21:2	165186783	GP	PS	0.01	0.04	1.71	0.01	0	1.67
Cer 18:1;2O/22:6	164247266	SP	Cer	0.04	0.14	1.43	0.02	0.05	1.54
PE O-21:0_13:1	164394718	GP	PE	0.01	0.04	1.7	0.01	0	1.65
5,7,4':Trihydroxyflavanone 7:sulfate	15460423	PK	PK	0.02	0.04	1.61	0.02	0	1.54
Cer 16:3;2O/36:10	164405304	SP	Cer	0.01	0.03	1.71	0.01	0	1.65
MGDG 23:0_14:1	138297017	GL	MGDG	0.02	0.08	1.65	0.01	0.01	1.66
PI 28:2;O2	LMGP20050001	GP	PI	0.02	0.02	1.69	0.01	0	1.65
PE:Cer 18:3;2O/18:4	165187687	SP	PE-Cer	0.01	0.03	1.72	0.01	0	1.67
DGGA 15:0_26:4	138301667	GL	DGGA	0.03	0.02	1.54	0.03	0.02	1.52
PA O-35:2	52929590	GP	PA	0.02	0.07	1.57	0.02	0.01	1.57
SM 14:3;2O/30:6	164193399	SP	SM	0.03	0.05	1.57	0.02	0.01	1.58
PS 41:7	138223286	GP	PS	0.01	0.06	1.68	0.01	0	1.69
LDGCC 28:6	164350874	GL	LDGCC	0.01	0.01	1.7	0.01	0	1.64
HexCer 34:3;O3	53355808	SP	HexCer	0.02	0.03	1.6	0.02	0	1.53
PG O-36:5	52927322	GP	PG	0.02	0.04	1.55	0.02	0.01	1.49
DGGA 13:0_20:0	138211085	GL	DGGA	0.03	0.14	1.5	0.01	0.01	1.62
HexCer 9:1;2O/42:8	164474301	SP	HexCer	0.01	0.02	1.72	0.01	0	1.69
PE 33:0_16:4	164421036	GP	PE	0.05	0.12	1.41	0.03	0.01	1.5
Cer 18:3;2O/42:10	164349319	SP	Cer	0.05	0.09	1.42	0.03	0.01	1.48
ST 24:1;O4;GlcA	195891	ST	ST	0.01	0.03	1.71	0.01	0	1.67
MGDG O-16:2_19:0	164207779	GL	MGDG	0.01	0.04	1.7	0.01	0	1.69
Gossypetin 7:methyl ether 8:acetate	44260028	PK	PK	0.01	0.02	1.73	0.01	0	1.69
NAGlySer 26:6/26:2	164472565	FA	NAGlySer	0.01	0.02	1.71	0.01	0	1.68
MGDG O-22:2_28:7	164446417	GL	MGDG	0.02	0.02	1.72	0.01	0	1.68
NAGly 24:3/24:4	164301579	FA	NAGly	0.03	0.15	1.49	0.01	0	1.67
PS O-18:2_26:0	165286386	GP	PS	0.01	0.03	1.73	0.01	0.01	1.69
HexCer 10:1;2O/44:9	164422317	SP	HexCer	0.02	0.02	1.7	0.01	0	1.66
SMGDG O-21:1_28:7	165193134	GL	SMGDG	0.01	0.01	1.72	0.01	0	1.66
NAGlySer 26:6/13:1	164505907	FA	NAGlySer	0.02	0.06	1.67	0.01	0	1.68
SHexCer 26:1;2O/24:2;O	164349519	SP	SHexCer	0.04	0.02	1.51	0.03	0	1.5
Cer 17	164344202	SP	Cer	0.02	0.09	1.61	0.01	0.03	1.63
PG O-20:3_26:1	165222721	GP	PG	0.01	0.02	1.73	0.01	0	1.69
PI 17:0_25:0	133033913	GP	PI	0.03	0.04	1.54	0.03	0.02	1.54
NAGlySer 24:6/26:1	164509591	FA	NAGlySer	0.02	0.05	1.68	0.01	0	1.69
PS O-22:3_25:0	165302935	GP	PS	0.01	0.03	1.72	0.01	0	1.69
HexCer 9:0;3O/27:0;(2OH)	164487376	SP	HexCer	0.01	0.03	1.72	0.01	0	1.69
TG 58:9	56938357	GL	TG	0.01	0.04	1.7	0.01	0	1.69
Pseudoecgonyl:CoA	71448901	FA	FA	0.02	0.05	1.69	0.01	0	1.7
Hex3Cer 36:9;2O	165328057	SP	Hex3Cer	0.02	0.02	1.67	0.01	0	1.63
PE O-18:0_20:3;O	138312495	GP	PE	0.01	0.05	1.69	0.01	0	1.69
DGGA 17:0_24:1	138125605	GL	DGGA	0.01	0.03	1.71	0.01	0	1.69
3':Hydroxy:3,5,8,4',5':pentamethoxy:6,7:methylenedioxyflavone	44260079	PK	PK	0.01	0.03	1.72	0.01	0	1.69
PI 16:0_20:5;4O	138304108	GP	PI	0.01	0.02	1.72	0.01	0	1.68
NAOrn 22:4/11:0	164465359	FA	NAOrn	0.01	0.09	1.76	0.01	0.07	1.74
PS 24:0_18:1	25245395	GP	PS	0.03	0.13	1.51	0.01	0	1.68
TG 57:9	56937923	GL	TG	0.01	0.02	1.73	0.01	0	1.69
PS O-25:0_26:4	165212475	GP	PS	0.01	0.03	1.72	0.01	0	1.69
PE:Cer 21:0;2O/20:2;O	165278328	SP	PE-Cer	0.01	0.04	1.69	0.01	0	1.68
TG 16:2_16:4_28:6	165293824	GL	TG	0.01	0.04	1.7	0.01	0	1.68
DGDG O-18:0_26:1	164400850	GL	DGDG	0.02	0.05	1.68	0.01	0	1.67
PE:Cer 12:0;2O/22:0;O	165335864	SP	PE-Cer	0.02	0.13	1.69	0.02	0.13	1.69
SM 50:12;3O	164506400	SP	SM	0.02	0.12	1.62	0.02	0.12	1.63
DGDG O-11:0_26:6	164487280	GL	DGDG	0.02	0.08	1.59	0.01	0	1.62
SHexCer 30:1;2O/24:2	138297699	SP	SHexCer	0.01	0.02	1.72	0.01	0	1.68
SL 19:3;O/24:0;O	164264194	SL	SL	0.01	0.06	1.69	0.01	0	1.7
HexCer 10:0;2O/44:10	164457039	SP	HexCer	0.01	0.01	1.74	0.01	0	1.68
MGDG 11:0_26:2	138126000	GL	MGDG	0.03	0.12	1.5	0.01	0	1.62

NAGlySer 22:0/20:4	164473045	FA	NAGlySer	0.01	0.02	1.73	0.01	0	1.67
CoA 14:4;O	LMFA07050380	FA	CoA	0.01	0	1.73	0.01	0	1.66
Cer 14	164230884	SP	Cer	0.02	0.07	1.57	0.01	0	1.58
SM 14:1;2O/26:1	164472876	SP	SM	0.04	0.17	1.43	0.01	0	1.66
HexCer 25:1;2O/18:5	164202766	SP	HexCer	0.01	0.03	1.7	0.01	0	1.67
Hex3Cer 16:0;2O/16:2	164225400	SP	Hex3Cer	0.04	0.03	1.44	0.04	0	1.41
DGDG 20:2_22:6	138304449	GL	DGDG	0.04	0.03	1.5	0.04	0	1.51
PI O-16:0_26:6	165199042	GP	PI	0.01	0.01	1.68	0.01	0	1.6
DGDG O-13:0_28:6	164311966	GL	DGDG	0.02	0.01	1.59	0.02	0	1.53
LDGTS 17:0	133017289	GL	LDGTS	0.02	0.06	1.65	0.01	0.01	1.66
ST 30:3;O5	45273389	ST	ST	0.02	0	1.65	0.02	0	1.59
HexCer 16:3;2O/30:2;2O	164464512	SP	HexCer	0.01	0.01	1.75	0.01	0	1.69
Cer 19:3;2O/38:10	164469215	SP	Cer	0.02	0.03	1.63	0.02	0	1.62
HBMP 16:3_22:6_22:6	138260988	GP	HBMP	0.03	0	1.55	0.03	0	1.5
DGDG O-16:1_28:0	164199480	GL	DGDG	0.01	0	1.75	0.01	0	1.69
HexCer 23:2;2O/24:6	164252392	SP	HexCer	0.01	0.01	1.75	0.01	0	1.69
PS O-20:2_26:1	165261960	GP	PS	0.01	0.01	1.75	0.01	0	1.69
PE:Cer 14:3;2O/26:6	165212135	SP	PE-Cer	0.01	0.01	1.72	0.01	0	1.66
Cer 14	164230884	SP	Cer	0.02	0.07	1.65	0.01	0	1.68
DGDG O-11:0_28:5	164465160	GL	DGDG	0.04	0	1.47	0.04	0	1.43
PE:Cer 26:2;2O/26:7	165190340	SP	PE-Cer	0.01	0	1.76	0.01	0	1.69
Hex2Cer 42:0;O2	LMSP0501AB18	SP	Hex2Cer	0.01	0	1.75	0.01	0	1.69
SL 15:3;O/26:7	164399916	SL	SL	0.04	0.02	1.44	0.04	0	1.37
NAGlySer 16:2/24:4	164492023	FA	NAGlySer	0.03	0.18	1.56	0.01	0.05	1.67
PMeOH 17:2_24:4	138251691	GP	PMeOH	0.01	0.01	1.73	0.01	0	1.66
PS O-15:0_26:4	165190931	GP	PS	0.01	0.01	1.74	0.01	0	1.67
MGDG O-24:2_28:7	164434792	GL	MGDG	0.01	0	1.74	0.01	0	1.68
CoA 6:1	163358979	FA	CoA	0.01	0	1.76	0.01	0	1.69
DG 14:0_20:1	138282457	GL	DG	0.02	0	1.62	0.02	0	1.56
DG O-21:0_14:1	164212284	GL	DG	0.02	0.1	1.59	0.01	0	1.66
PS 3:0_16:2	138260835	GP	PS	0.01	0	1.72	0.01	0	1.63
SM 10:0;2O/28:0	164396151	SP	SM	0.02	0.05	1.65	0.01	0	1.63
SM 18:1;2O/34:8	164410511	SP	SM	0.01	0.01	1.75	0.01	0.01	1.67
MGDG 24:4_26:4	138132486	GL	MGDG	0.04	0.01	1.49	0.04	0.01	1.46
Ganocasurarin A	156582628	PR	PR	0.05	0	1.45	0.05	0	1.43
HBMP 16:3_16:4_20:3	138199135	GP	HBMP	0.01	0.02	1.71	0.01	0	1.67
PG 18:0_18:0;O	138168075	GP	PG	0.02	0	1.67	0.02	0	1.59
PG O-26:0_26:6	165331750	GP	PG	0.01	0	1.75	0.01	0	1.68
DGDG O-22:1_23:0	164422245	GL	DGDG	0.01	0	1.76	0.01	0	1.69
PIM2 36:2	LMGP15010073	GP	PIM2	0.01	0	1.76	0.01	0	1.69
Apigenin 7:rhamnoside:4':rutinoside	163011040	PK	PK	0.01	0	1.76	0.01	0	1.69
FA 26:4	165283605	FA	FA	0.01	0.05	1.71	0.01	0.01	1.69
MGDG O-22:0_28:6	164384720	GL	MGDG	0.01	0	1.75	0.01	0	1.69
ADGGA 22	138196138	GL	ADGGA	0.01	0	1.75	0.01	0	1.68
PA 17:2_18:5	138286781	GP	PA	0.01	0	1.75	0.01	0	1.69
SM 10:1;2O/34:9	164229071	SP	SM	0.03	0	1.54	0.03	0	1.48
PI:Cer 22:3;2O/26:0;O	165317787	SP	PI-Cer	0.01	0	1.75	0.01	0	1.69
SM 10:1;2O/42:9	164278400	SP	SM	0.05	0	1.45	0.05	0	1.43
SL 21:0;O/26:3	164425253	SL	SL	0.05	0	1.4	0.05	0	1.31
MGDG O-16:3_28:6	164397582	GL	MGDG	0.04	0	1.46	0.04	0	1.42
Cer 15	164412894	SP	Cer	0.03	0	1.55	0.03	0	1.52
NAOrn 26:7/22:4	164507812	FA	NAOrn	0.03	0.02	1.51	0.03	0	1.47
LDGTS 20:5	138274914	GL	LDGTS	0.02	0.01	1.65	0.02	0	1.61
PS O-22:0_20:1	165285753	GP	PS	0.02	0.05	1.66	0.01	0	1.67
alpha,alpha':Trehalose 4,6:dioleate	LMSL03001273	SL	SL	0.02	0.01	1.57	0.02	0	1.48
DLCL 24:0_16:2	165205240	GP	DLCL	0.02	0	1.66	0.02	0	1.59
SM 42:1;3O	164377505	SP	SM+O	0.01	0.01	1.74	0.01	0	1.66
MGDG O-11:0_28:1	164238376	GL	MGDG	0.01	0.01	1.74	0.01	0	1.67
10S,11R:dichlorO-7,11:dimethyl:3:methylene:4R:hydroxy:6E,8E,12:tridecatrienoic acid	21632532	PR	PR	0.05	0	1.45	0.05	0	1.36
PI O-25:0_26:5	165276954	GP	PI	0.03	0.01	1.52	0.03	0	1.5
HexCer 40:0;2O	164224204	SP	HexCer-NDS	0.01	0.01	1.73	0.01	0	1.67

MGDG O-24:4_22:5	164194992	GL	MGDG	0.03	0.01	1.54	0.03	0	1.5
MGDG O-28:7_20:0	164289169	GL	MGDG	0.03	0.01	1.55	0.03	0	1.5
1':Hydroxy:4:ketO-gamma:carotene glucoside hexadecanoate	101534475	PR	PR	0.02	0.01	1.64	0.02	0	1.6
HexCer 28:3;3O(FA 17:2)	165292263	SP	HexCer	0.02	0.02	1.59	0.02	0	1.55
Myricetin 3,7:diglucuronide	102274234	PK	PK	0.03	0.09	1.56	0.02	0.03	1.53
BMP 22:1_22:4	138245890	GP	BMP	0.03	0	1.57	0.03	0	1.53
DG 22:3_34:9	164291440	GL	DG	0.04	0.01	1.4	0.04	0	1.33
p:coumaroyl:homoserine lactone	71311837	FA	FA	0.04	0.05	1.5	0.03	0	1.49
LNAPE 22:2/N:23:0	165286777	GP	LNAPE	0.01	0	1.74	0.01	0	1.67
18:bromO-octadecanoic acid	5312948	FA	FA	0.01	0	1.72	0.01	0	1.64
MGDG O-28:5_22:3	164213344	GL	MGDG	0.05	0.01	1.44	0.04	0	1.43
DGDG O-16:4_4:0	164434480	GL	DGDG	0.05	0	1.42	0.05	0	1.32
SMGDG O-24:4_2:0	165278327	GL	SMGDG	0.04	0	1.5	0.04	0	1.4
DGDG 25:0_17:2	138140439	GL	DGDG	0.02	0.01	1.66	0.02	0	1.62
MGDG O-22:4_28:3	164255113	GL	MGDG	0.02	0.02	1.64	0.02	0	1.59
HexCer 18:1;2O/24:5	164384224	SP	HexCer	0.03	0.04	1.51	0.02	0	1.51
MGMG 16:2	LMGL04010008	GL	MGMG	0.04	0.03	1.44	0.04	0	1.44
MGDG 24:1_26:4	138183244	GL	MGDG	0.02	0.03	1.58	0.02	0	1.57
PI O-23:0_26:0	165218719	GP	PI	0.04	0.03	1.45	0.04	0	1.45
PI O-24:2_26:7	165269073	GP	PI	0.04	0.01	1.49	0.04	0	1.49
FAHFA 20:3/26:0	138260571	FA	FAHFA	0.02	0.22	1.6	0.03	0.27	1.36
FOH 14:0	8209	FA	FOH	0.02	0.09	1.56	0.01	0	1.65
BMP 23:0_22:5	138301187	GP	BMP	0.04	0.12	1.37	0.02	0	1.54
BMP 27:0_20:5	138231287	GP	BMP	0.03	0.13	1.43	0.01	0	1.63
PG O-17:0_26:3	165266759	GP	PG	0.03	0.06	1.5	0.02	0	1.53
SM 42:3;2O	164243513	SP	SM	0.04	0.1	1.43	0.02	0	1.52
SQDG 20:0_18:2	138117475	GL	SQDG	0.03	0.03	1.51	0.02	0	1.46
MGDG O-20:0_28:7	164237037	GL	MGDG	0.04	0.09	1.43	0.02	0	1.52
BMP 21:0_26:4	138208293	GP	BMP	0.02	0.02	1.65	0.01	0	1.63
HexCer 23:1;2O/20:3	164484573	SP	HexCer	0.02	0.06	1.57	0.02	0	1.61
HexCer 16:2;2O/31:1;O	164422643	SP	HexCer	0.04	0.01	1.46	0.04	0	1.44
ST 29:0;O3	3036251	ST	ST	0.04	0.06	1.45	0.03	0	1.51
8:Hydroxyapigenin 8:(2'',4'':disulfatoglucuronide)	5318665	PK	PK	0.02	0.02	1.7	0.02	0	1.66
Hex2Cer 16:0;2O/16:1	164401774	SP	Hex2Cer	0.03	0.01	1.47	0.03	0	1.39
DG 16:2_40:11	164342974	GL	DG	0.02	0.03	1.64	0.02	0	1.59
NAOrn 16:2/26:1	164307463	FA	NAOrn	0.03	0.04	1.51	0.02	0	1.53
HexCer 26:0;2O/26:5	164226371	SP	HexCer	0.02	0.02	1.71	0.01	0	1.68
HexCer 29:0;2O/20:4	164406935	SP	HexCer	0.03	0.11	1.51	0.01	0.01	1.65
Cer 8:0;3O/38:0;(2OH)	164219685	SP	Cer	0.03	0.11	1.52	0.01	0	1.67
SL 22:3;O/24:2;O	164205863	SL	SL	0.02	0	1.66	0.02	0	1.61
DG O-28:7_28:7	164294319	GL	DG	0.01	0.04	1.67	0.01	0	1.67
SMGDG O-13:0_28:2	165216205	GL	SMGDG	0.02	0.02	1.61	0.02	0	1.56
Isoscutellarein 4':methyl ether 8:(2'',4'':disulfatoglucuronide)	5318670	PK	PK	0.02	0.07	1.58	0.01	0	1.63
BMP 24:0_26:4	138142843	GP	BMP	0.02	0.08	1.55	0.01	0	1.64
FA 22:7;O2	131840732	FA	FA	0.03	0.38	1.63	0.01	0.31	1.7
BMP 5:0_7:0	138141350	GP	BMP	0.03	0.37	1.57	0.01	0.2	1.71
ST 24:6;O5	5284110	ST	ST	0.02	0.41	1.6	0.01	0.27	1.68
DGDG O-17:2_9:0	164451014	GL	DGDG	0.02	0.12	1.57	0.01	0.11	1.57
Austrobuxusin K	154585435	PR	PR	0.03	0.3	1.55	0.01	0.14	1.66
SM 17:3;2O/3:0	164301546	SP	SM	0.04	0.14	1.45	0.04	0.12	1.37
ST 23:4;O6	164613318	ST	ST	0.02	0.26	1.61	0.01	0.13	1.73
Nitiducol	5378475	PK	PK	0.02	0.14	1.5	0.02	0.08	1.49
Phytyl diphosphate(1)	14556929	PR	PR	0.04	0.35	1.44	0.02	0.23	1.66
5,7:Dimethoxy:8:prenylflavan	44257186	PK	PK	0.02	0.23	1.61	0.02	0.15	1.59
Uscharidin	441874	ST	ST	0.02	0.11	1.54	0.02	0.06	1.47
BMP 25:0_26:4	138220416	GP	BMP	0.01	0.01	1.72	0.01	0	1.65
ADGGA 20	138206483	GL	ADGGA	0.02	0.02	1.65	0.01	0	1.6
Cer 23:2;2O/40:8	164198423	SP	Cer	0.02	0.02	1.64	0.02	0.02	1.6
MGDG O-24:0_28:7	164254044	GL	MGDG	0.02	0.01	1.61	0.02	0	1.57
LDGCC 40:9	164371317	GL	LDGCC	0.01	0	1.76	0.01	0	1.69
HexCer 10:0;2O/42:1	164287587	SP	HexCer	0.02	0.02	1.64	0.02	0.03	1.6
HexCer 31:0;2O/22:5	164476811	SP	HexCer	0.01	0.06	1.67	0.01	0.04	1.66

HexCer 15:1;2O/40:5	164473229	SP	HexCer	0.01	0.05	1.67	0.01	0.05	1.66
DGDG 13:0_27:0	138188075	GL	DGDG	0.03	0	1.52	0.03	0	1.42
PG O-26:0_20:2	165317269	GP	PG	0.02	0	1.67	0.02	0	1.58
TG 53:4	56937354	GL	TG	0.01	0	1.73	0.01	0.04	1.62
HexCer 23:3;2O/32:2	164285915	SP	HexCer	0.01	0.07	1.71	0.02	0.15	1.57
Cer 22	164385781	SP	Cer	0.03	0	1.53	0.03	0.02	1.45
DG 17:2_42:10	164216726	GL	DG	0.03	0	1.57	0.03	0.01	1.47
Cer 23:2;2O/42:8	164278962	SP	Cer	0.02	0	1.67	0.02	0.02	1.6
PI:Cer 48:3;3O	164227319	SP	PI-Cer	0.02	0	1.67	0.02	0.01	1.6
HexCer 35:0;3O/13:0;(2OH)	164484458	SP	HexCer	0.04	0	1.51	0.04	0	1.43
SM 31:2;2O/20:0	164225597	SP	SM	0.01	0	1.73	0.02	0.05	1.63
NAOrn 18:5/21:0	164498444	FA	NAOrn	0.02	0	1.66	0.02	0.04	1.51
TG 56:5	56937445	GL	TG	0.01	0	1.72	0.02	0.06	1.58
DG 15:1_44:10	164253521	GL	DG	0.02	0	1.61	0.03	0.04	1.47
TG 16:0_17:0_22:5	138206196	GL	TG	0.02	0	1.6	0.03	0.06	1.44
HexCer 13:0;2O/42:4	164190451	SP	HexCer	0.01	0	1.74	0.04	0.2	1.46
7,8,4':Trihydroxy:3',5':di-methoxyflavanone 4':O-gluco-side	42607837	PK	PK	0.04	0	1.49	0.05	0.03	1.39
DG 22:4_40:7	164259647	GL	DG	0.04	0	1.5	0.04	0.03	1.39
HexCer 31:0;2O/22:6	164411441	SP	HexCer	0.02	0	1.64	0.02	0.04	1.51
NAOrn 16:1/22:5	164438516	FA	NAOrn	0.04	0	1.46	0.05	0.04	1.32
Cer 15	164412894	SP	Cer	0.04	0	1.51	0.05	0.04	1.4
HexCer 28:4;3O(FA 18:1)	165306670	SP	HexCer	0.01	0	1.72	0.02	0.05	1.59
DG 15:1_44:9	164387657	GL	DG	0.02	0	1.64	0.05	0.17	1.37
TG 10:0_22:2_30:4	164367224	GL	TG	0.01	0	1.74	0.04	0.19	1.45
HexCer 19:3;2O/44:11	164262437	SP	HexCer	0.04	0	1.53	0.04	0.03	1.41
TG 18:0_18:1_19:2	165260394	GL	TG	0.01	0	1.75	0.03	0.18	1.52
DG 16:3_42:6	164412059	GL	DG	0.02	0	1.62	0.04	0.13	1.42
HexCer 17:0;2O/44:1	164262164	SP	HexCer	0.02	0	1.6	0.02	0	1.52
DGCC 21:0_43:0	164276395	GL	DGCC	0.02	0	1.64	0.02	0	1.57
DGCC 21:0_44:0	164205232	GL	DGCC	0.02	0	1.61	0.02	0	1.57
SM 32:2;2O/38:6	164483228	SP	SM	0.02	0.05	1.62	0.04	0.11	1.5
DG 12:0_24:1	138235455	GL	DG	0.05	0	1.41	0.05	0.01	1.31
TG 10:0_13:1_31:0	164293120	GL	TG	0.01	0	1.72	0.01	0	1.65
R:Oblatone A	LMPK13070005	PK	PK	0.03	0.25	1.55	0.01	0.03	1.66
FA 28:8;O5	11038270	ST	FA	0.02	0.25	1.53	0.03	0.27	1.51
Patuletin 3:glucoside:7:sulfate	101421040	PK	PK	0.02	0.22	1.52	0.03	0.26	1.54
SMGDG O-10:0_10:0	165271414	GL	SMGDG	0.03	0.2	1.51	0.03	0.22	1.49
SMGDG O-22:6_4:0	165194863	GL	SMGDG	0.03	0.11	1.47	0.02	0.03	1.51
FAHFA 4:0/17:1	138166043	FA	FAHFA	0.05	0.3	1.29	0.01	0.14	1.58
DGDG 2:0_8:0	138128354	GL	DGDG	0.04	0.04	1.32	0.05	0.06	1.18
N:jasmonoyl:dopamine	11660229	FA	FA	0.02	0.19	1.53	0.02	0.16	1.56
manumycin	6438330	FA	FA	0.03	0.14	1.43	0.02	0.05	1.46
PA 6:0_22:6	138201824	GP	PA	0.02	0.2	1.54	0.01	0.08	1.69
CerP 19:0;2O/2:0	164220724	SP	CerP	0.02	0.19	1.55	0.01	0.1	1.62
Heteranthin	16083083	PR	PR	0.03	0.17	1.49	0.02	0.08	1.54
ST 28:6;O3	10251684	ST	ST	0.02	0.2	1.52	0.01	0.14	1.57
6:Methoxykaempferol 3,7:bis(3:acetylrhamnoside)	14502653	PK	PK	0.02	0.25	1.56	0.04	0.36	1.29
(1S):1:hydroxy:23,24:didehy-drO-25,26,27:trinorcalciol	17756764	ST	ST	0.03	0.26	1.54	0.01	0.15	1.52
LDGCC 34:3	164238901	GL	LDGCC	0.02	0.26	1.48	0.01	0.13	1.65
LPA 24:1	138141870	GP	LPA	0.04	0.3	1.39	0.02	0.21	1.55
PA 7:0_22:6	138241050	GP	PA	0.04	0.13	1.39	0.03	0.09	1.39
DG O-15:1_3:0	164240649	GL	DG	0.03	0.3	1.35	0.01	0.17	1.56
FOH 19:4;O3	6710762	FA	FOH	0.04	0.3	1.38	0.04	0.3	1.44
LPA 15:0	17786738	GP	LPA	0.02	0.21	1.51	0.01	0.13	1.57
BMP 18:3_18:5	138152180	GP	BMP	0.03	0.23	1.46	0.01	0.13	1.54
SMGDG O-10:0_7:0	165243809	GL	SMGDG	0.04	0.15	1.41	0.03	0.11	1.38
SPB 27:0;2O	164430987	SP	SPB	0.03	0.2	1.47	0.01	0.04	1.74
LNAPS 4:0/N:16:2	165297097	GP	LNAPS	0.04	0.03	1.41	0.03	0	1.35
steviol:19:O-beta:D:glucoside	152758	PR	PR	0.02	0.06	1.56	0.03	0.07	1.51
Gibberellin A36	443455	PR	PR	0.02	0.14	1.55	0.01	0.05	1.67
HexCer 16:1;2O/3:0	164376142	SP	HexCer	0.02	0.21	1.5	0.01	0.1	1.62
N:decanoyl histidine	22804201	FA	FA	0.02	0.02	1.59	0.02	0.04	1.52

HexCer 20:1;2O/44:12	164213461	SP	HexCer	0.02	0.01	1.62	0.02	0.02	1.52
AHexCer (O-26		SP	AHexCer	0.02	0	1.57	0.02	0.01	1.46
DG O-22:1_14:1	164467845	GL	DG	0.02	0.01	1.63	0.02	0.01	1.53
DGTS 18:0_15:1	138307779	GL	DGTS	0.03	0	1.54	0.03	0.01	1.43
ST 28:1;O2	52931372	ST	ST	0.05	0	1.4	0.05	0	1.29
LDGTS 17:2	138183223	GL	LDGTS	0.02	0.04	1.58	0.02	0.06	1.5
HexCer 14:2;2O/8:0	164265491	SP	HexCer	0.01	0.11	1.84	0.03	0.22	1.6
PEtOH 16:0_26:4	138244092	GP	PEtOH	0.04	0	1.49	0.04	0.02	1.38
SL 24:0;O/25:0;O	164377055	SL	SL	0.01	0.03	1.69	0.01	0.01	1.64
2:deoxy:20:hydroxyecdysone 22:phosphate	21122088	ST	ST	0.02	0.04	1.6	0.02	0.03	1.56
SL 18:3;O/26:5	164281705	SL	SL	0.04	0.07	1.47	0.05	0.1	1.4
PC 16:0_24:6	164408413	GP	PC	0.03	0.03	1.55	0.03	0.04	1.47
LNAPE 13:1/N:22:6	165224529	GP	LNAPE	0.03	0	1.5	0.04	0.08	1.33
1:hexadecanyl:2:((2':α:glucosyl):β:glucosyl):3:β:xylosyl:sn:glycerol	LMGL04010001	GL	GL	0.04	0.01	1.43	0.05	0.05	1.3
BMP 7:0_20:1	138127284	GP	BMP	0.02	0.01	1.6	0.02	0	1.53
